# Association between microRNAs 10b/21/34a and acute toxicity in glioblastoma patients treated with radiotherapy and temozolomide

**DOI:** 10.1038/s41598-022-11445-9

**Published:** 2022-05-07

**Authors:** Aleksandar Stepanović, Marina Nikitović, Tatjana P. Stanojković, Danica Grujičić, Zoran Bukumirić, Ivana Srbljak, Rosanda Ilić, Snežana Milošević, Tatjana Arsenijević, Nina Petrović

**Affiliations:** 1 Department of Radiation Oncology, Institute for Oncology and Radiology of Serbia, Belgrade, Serbia; 2 Faculty of Medicine, University of Belgrade, Belgrade, Serbia; 3 Department of Experimental Oncology, Institute for Oncology and Radiology of Serbia, Belgrade, Serbia; 4 Clinic of Neurosurgery, Neuro-Oncology Department, University Clinical Center of Serbia, Belgrade, Serbia; 5 Institute for Medical Statistics and Informatics, Faculty of Medicine, University of Belgrade, Belgrade, Serbia; 6 “VINČA” Institute of Nuclear Sciences-National Institute of the Republic of Serbia, University of Belgrade, Belgrade, Serbia

**Keywords:** CNS cancer, Molecular biology, Oncology

## Abstract

A personalized approach to chemoradiation is important in reducing its potential side effects and identifying a group of patients prone to toxicity. MicroRNAs have been shown to have a predictive potential for radiotoxicity. The goal of the study was to test if levels of miRNA in peripheral blood mononuclear cells of glioblastoma patients are associated with toxicity and to identify the peak time point for toxicity. MicroRNA-10b/21/34a levels were measured in 43 patients with and without toxicity, at baseline, at the 15th, and at the 30th fraction by Real-Time quantitative Polymerase Chain Reaction. MicroRNA-10b/21 levels increased with toxicity grade (p = 0.014; p = 0.013); miR-21/34a levels were significantly different between patients with and without toxicity at the 15th fraction (p = 0.030; p = 0.045), while miR-34a levels significantly changed during treatment (p < 0.001). All three miRNAs showed a significantly high positive correlation with one another. MiR-34a might be considered as a predictive factor for toxicity due to its changes during treatment, and differences between the groups with and without toxicity; miR-10b might be used to predict toxicity; miR-10b/21 might be used for predicting the grade of toxicity in GB patients.

## Introduction

Glioblastoma (GB) is the most frequent primary malignant brain tumor in adults, infiltrative, and highly aggressive^1^. Despite all treatment modalities, survival rates are still low, and the disease-free survival is short. Current standard therapy for GB patients involves surgical resection and postoperative radiotherapy (RT) with concomitant and adjuvant temozolomide (TMZ)^1^. The introduction of TMZ in multimodality therapy has improved treatment outcomes and has been associated with a 2-year survival rate of 27.2%^2^. High infiltrative potential frequently leads to chemo-radio resistance, and the 5-year overall survival rates are around 10%^3,4^. Numerous studies have focused on identifying molecular characteristics associated with the molecular basis underlying GB pathology, such as isocitrate dehydrogenase mutation (IDH) status, loss of phosphatase, and tensin homolog (PTEN) heterozygosity, amplification of epidermal growth factor receptor vIII (EGFRvIII)^5,6^. Furthermore, the methylation status of O^6^‐methylguanine‐DNA methyltransferase (MGMT), and expression level changes of miRNA molecules were shown to be associated with GB pathology and prognosis, as well^7,8^.

MicroRNA (miRNAs) are small non-coding transcriptome elements that cause translational repression of target messenger RNA (mRNA), resulting in reduced protein synthesis. MicroRNA level changes are associated with GB tumor stage, type, and subtype, response to therapy, overall survival, and prognosis^9,10^. Some microRNAs have response to radiation treatment, they can be utilized as radioprotectors, or to improve sensitivity to therapy^11^. For example, miR-26a/124/128/145/221/222/590 have been shown to enhance radiosensitivity of glioblastoma cells, while miR-135b/21/210/212 lowered sensitivity of GB cells to irradiation^11^. Moreover, miR-34a reverses TMZ resistance of GB cells^12^, while miR-132 induced resistance to TMZ in U87MG primary Gb cell line^13^.

Although microRNAs with the ability to modulate tumor response to chemo-RT are being investigated, biomarkers for prediction of acute toxicity and an increased risk of adverse effects still have not been identified. Effects of acute radiation toxicity emerge during RT or up to 3 months after the treatment, and they are induced by multiple cellular and molecular mechanisms. Factors correlating with CNS radiation toxicity include injury to vessel structures, deletion of oligodendrocyte-2 astrocyte progenitors, deletion of specific neural stem populations, and modification of cytokine expression^14^. The acute adverse events are primarily related to brain edema. Brain edema may occur at the very beginning of radiation treatment and after one fraction of 200 rads (2 Gy)^15^. Late toxicity induced by radiotherapy develops from 3 months^16^ up to a couple of years after the treatment and includes diffuse leukoencephalopathy (fatigue, mental changes, memory loss, dementia), or focal radionecrosis. Treatment-related factors such as dose per fraction, irradiated volume, number of fractions, total received/absorbed dose, and exposure duration are not sufficient to predict normal-tissue reaction^17^. Tests predicting the risk of side effects following RT which involve genetic background, transcriptome, and proteome changes could be used to individualize radiotherapy schedules to reduce the doses in sensitive patients, to increase the quality of life of cancer patients. A test that predicts a low risk of side effects can also be used to help clinicians to intensify treatments, and to single out candidates for dose escalation to increase survival chances. It has also been proposed that microRNAs might serve as factors for stratifying patients into groups with high or low risk for developing side effects and acute/late radio-chemotoxicity^18^.

Peripheral blood mononuclear cells (PBMCs) also synthesize particular miRNA molecules, in response to ionizing radiation^19^. MicroRNA molecules enter PBMCs and lymphocytes of peripheral blood from the circulation via miRNA trafficking^20,21^. For example, miR-21 is involved in bystander effects^22^ meaning that irradiated cells transfer signals about exposure to radiation to the surrounding non-irradiated cells^23^. Also, RT induces a DNA damage response in PMBCs^24^. MicroRNAs modulate cell response to ionizing radiation, through the change of translation intensity genes associated with DNA damage repair^22^. According to recent studies, miR-10b is described as a potential predictive parameter for response to radiotherapy in GB patients^25^. An increase in miR-10b has been associated with lower levels of radiation-induced apoptosis, by regulating the components of the protein kinase B (AKT) signaling pathway, which promotes the processes of cancer cell invasion and migration^25^. Co-inhibition of microRNA 10b and miR-21 have been shown to have a synergistic effect in reducing the proliferation and invasion of glioma cancer cells^26^. MicroRNA 34a has also been shown to regulate radiation response in many different types of tumors including GB^27,28^. Micro RNA-21 is the most frequently investigated miRNA in cancer, in general with relatively high oncogenic potential, and influences on response to radiation treatment. It has been shown that the elevated level of miR-21 is connected with a resistance of glioma cells to radiation. Overexpression of miR-21 is associated with a poor prognosis; invasiveness and poorer response to radiotherapy^29,30^. It has been observed that levels of miR-21 significantly changed during radiation treatment and differed among patients with prostate cancer with and without acute genitourinary radiotoxicity^31^.

The main goal of this research is to investigate the potential associations between miR-10b/21/34a expression levels and a grade of acute toxicity. The second goal is to see how levels of the three miRNAs change in peripheral blood mononuclear cells (PBMCs) of GB patients during radio-chemotherapy at particular time points at baseline, at the 15th fraction of RT, and at the last fraction of RT-30th fraction. The third goal is to compare the changes in systemic miRNA levels in GB patients who developed acute toxicity and patients who did not develop toxicity, to see if any miRNA has the potential to be studied in the future as an additional factor, a future biomarker of toxicity, and to identify time-point(s) with significant differences. The fourth goal is to see if miRNA levels correlate with one another to see if they could act combinatorically in response to RT in patients with and without toxicity. Additionally, we aimed to discover potentially shared target genes for miR-10b/21/34a by miRNet^32^ online software, and to describe molecules of signaling pathways activated in radiation response.

## Results

In this research, we have investigated miR10b/21/34a expression levels in 43 patients at three time points (129 samples for each miRNA molecule); at baseline, at the 15th fraction of radiation treatment, and at the last fraction of radiotherapy (30th fraction). Twenty-one did not have any side effects of RT, while 22 patients had grade 1, 2 or grade 3 toxicity at either 15th or 30th fraction. Histological subtypes analyzed in this cohort were presented in Table 1.

**Table 1:** Patients' clinical characteristics: differences between groups of patients with and without toxicity-description of groups.

Patients' clinical characteristics	Frequency (percent) of patients per group/mean ± standard deviation	Differences between groups, p values
**Gender**		
Female without toxicty	6 (28.6%)	0.817
Female with toxicity	7 (31.8%)	
Male without toxicity	15 (71.4%)	
Male with toxicity	15 (68.2%)	
**Age at diagnosis**		
Without toxicity	56.57 ± 8.060	0.736
With toxicity	57.68 ± 12.748	
Patients without toxicity	21 (48.8%)	
**Patients experienced toxicity (any time)**	22 (51.2%)	
Toxicity in 15th fraction	15 (68.2%)	
No toxicity in 15th fraction	7 (31.8%)	
Toxicity in 30th fraction	17 (77.3%)	
No toxicity in 30th fraction	5 (22.7%)	
**Toxicity grade 15th fraction**		
1	7 (46.7%)	
2	6 (40%)	
3	2 (13.3%)	
**Toxicity grade 30th fraction**		
1	5 (29.4%)	
2	9 (52.9%)	
3	3 (17.6%)	
**IDH mutation status**		
Mutant without toxicity	1 (4.8%)	0.016
Mutant with toxicity	1 (4.5%)	
Wild-type without toxicity	15 (71.4%)	
Wild-type with toxicity	7 (31.8%)	
NOS without toxicity	5 (23.8%)	
NOS with toxicity	14 (63.6%)	
**Histological subtype**		
Glioblastoma without toxicity	21 (100%)	0.167
Glioblastoma with toxicity	18 (81.8%)	
Glioblastoma with primitive neuronal component without toxicity	0 (0.0%)	
Glioblastoma with primitive neuronal component with toxicity	1 (4.5%)	
Giant cell glioblastoma without toxicity	0 (0.0%)	
Giant cell glioblastoma with toxicity	3 (13.6%)	

Age at diagnosis was presented as means ± standard deviation. *NOS* not otherwise specified.

Groups of patients with and without toxicity did not statistically differ in gender frequency distribution and mean age, according to Pearson Chi-Square test, p = 0.817, and Student’s t-test, p = 0.736, respectively (Table 1). Frequency distribution of patients divided into groups according to IDH mutation status has showed that a significantly higher number of patients without toxicity was IDH wild-type, while patients with toxicity were predominantly distributed in “not otherwise specified” (NOS) group (p = 0.016, Fisher’s exact test, Table 1).

**Figure 1:**
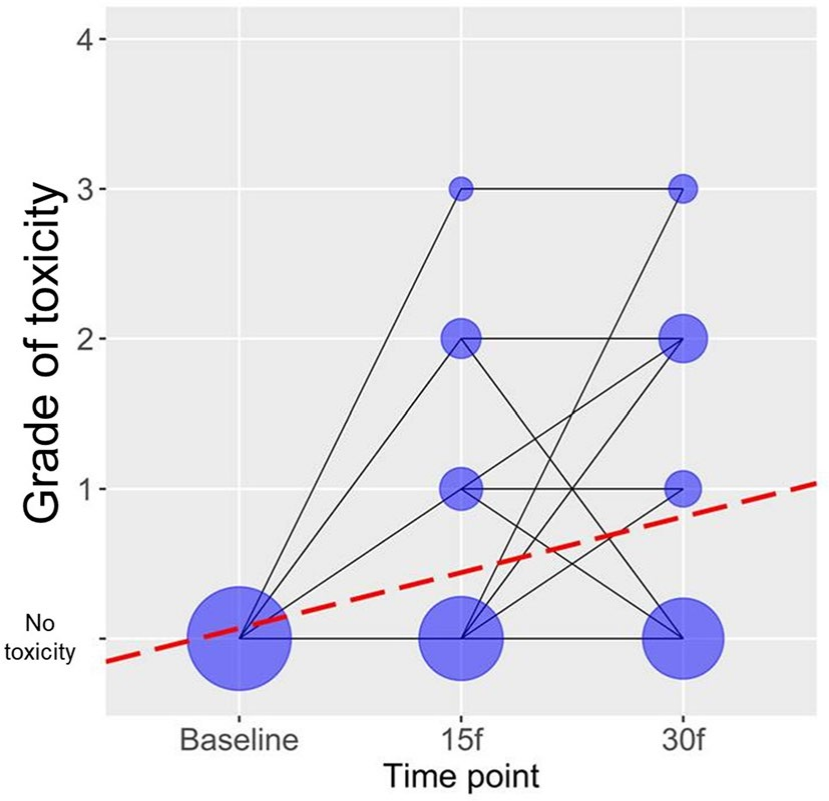
Grade of toxicity over time during treatment with RT and TMZ. “No toxicity” represents patients at baseline, before treatment. It does not represent grade. The grade of toxicity was defined as 1, 2, 3, and 4 (in our cohort there were no patients with grade 4 toxicity, although the maximal grade according to CTCAE ver.5.0 scoring is 4). *According to CTCAE ver. 5.0 grade 5 (death) is not adequate for some adverse events and therefore was not an option.

According to the Mixed-effect linear model, there has been a statistically significant increase in the toxicity grade over time, during radiation therapy with TMZ (b = 0.372, p < 0.001, Fig. 1). Furthermore, individual miRNAs have been included in additional univariate analysis, with each miRNA analyzed separately. The three miRNAs have not been taken into consideration in the multivariate model, because of their multicollinearity (high positive correlations among one another at all time points). Univariate analysis has showed that higher levels of miR-10b and miR-21 are predominantly associated with a higher grade of toxicity, respectively (b = 0.0006, p = 0.014; b = 0.0008, p = 0.013, Table 2). MicroRNA-34 levels have not been significantly associated with toxicity grades (b = 0.0005, p = 0.400, Table 2).

**Table 2:** Univariate analysis-miR-10b/21/34a and toxicity grade.

MicroRNA	Time point			Univariate analysis^a^	
	Baseline	15th f	30th f	b	*p*-value
miR-10b	111.28 (2.13–816.89)	92.99 (1.00–922.88)	117.78 (1.47–2751.50)	0.0006	**0.014**
miR-21	62.38 (2.68–825.43)	50.53 (2.79–960.07)	61.56 (1.00–1940.21)	0.0008	**0.013**
miR-34a	12.05 (1.00–210.55)	37.35 (3.48–352.38)	82.94 (2.94–871.28)	0.0005	0.400

p values equal or less than 0.05 are significant (bold).

^a^Univariate ordinal regression models with the degree of toxicity as dependent variable. Median values of microRNA expression values with min–max ranges in parentheses were presented for each time point-at baseline, 15th fraction (15th f) and 30th fraction (30th f).

**Figure 2:**
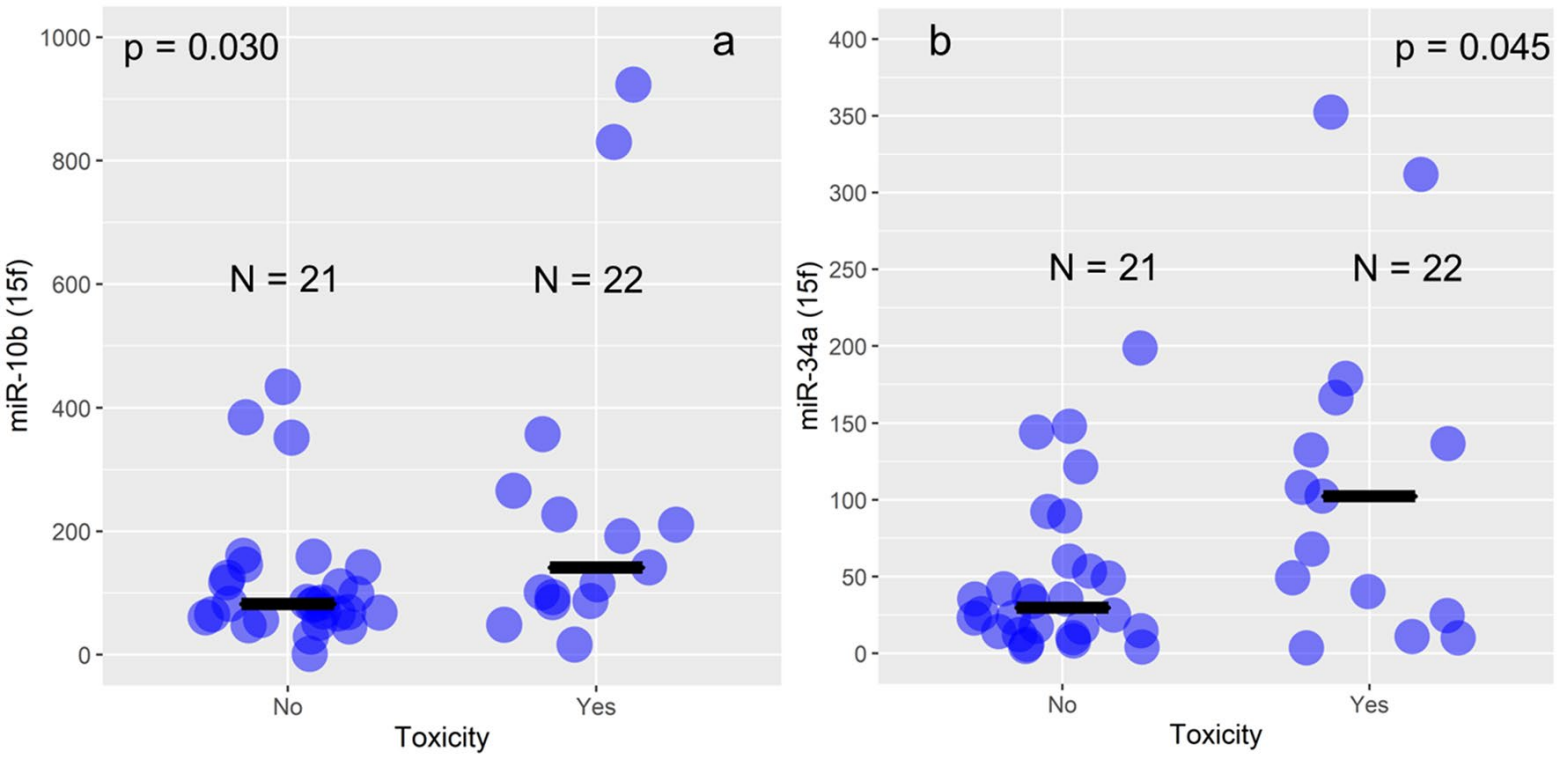
Comparison between patients with and without side effects at the 15th fraction of RT. Violet dots represent each patient, while the black line represents the median value of miR-10b (**a**) and miR-34a (**b**) expression values (*RQ* relative quantity units) at each analyzed group-with and without side effects at the 15th fraction-15f).

The juxtaposition between the groups without and with toxicity at 15th fraction point has shown significantly higher levels of miR-10b and miR-34a levels in groups with toxicity compared with groups without toxicity at that particular point, respectively (p = 0.030, and p = 0.045, Mann–Whitney U test, Fig. 2, Table 3), while at baseline and at 30th fraction, there were no differences between the groups.

**Table 3:** Differences in miR-10b/21/34a relative expression levels between the patients without and with toxicity within GB patients, at Baseline, the 15th, and the 30th fraction of radiation therapy.

	Without toxicity	With toxicity	p values with vs without toxicity
**MiR-10b relative expression levels**^a^			
Baseline	98.43 (19.24–622.53)	147.51 (2.13–816.89)	
15th fraction	84.62 (29.22–433.53)	95.47 (1.00–922.88)	**0.030**
30th fraction	145.71 (1.47–631.66)	112.95 (14.59–2751.50)	0.747
p value baseline vs 15th f vs 30th f	0.953	0.834	
**MiR-21 relative expression levels**^a^			
Baseline	40.84 (5.82–278.98)	73.48 (2.68–825.43)	
15th fraction	30.80 (4.37–542.32)	80.39 (2.79–960.07)	0.108
30th fraction	52.35 (1.00–340.62)	68.53 (7.86–1940.21)	0.882
p value baseline vs 15th f vs 30th f	0.229	0.142	
**MiR-34a relative expression levels**^a^			
Baseline	10.05 (1.55–105.20)	15.81 (1.00–210.55)	
15th fraction	25.72 (5.78–198.64)	63.92 (3.48–352.38)	**0.045**
30th fraction	49.25 (2.94–871.28)	89.44 (7.51–853.35)	0.941
p value baseline vs 15th f vs 30th f	**p < 0.001**	**p < 0.001**	

^a^Median values of relative miR-10b/21/34a expression with minimum and maximum in parentheses. p values equal or less than 0.05 were considered significant according to the result of Wilcoxon’s signed rank test (between 2 groups) and Friedman’s test for 3 groups comparisons (in bold style). p values between 0.1 and 0.05 were considered as statistical trend, presented in bold style.

**Figure 3:**
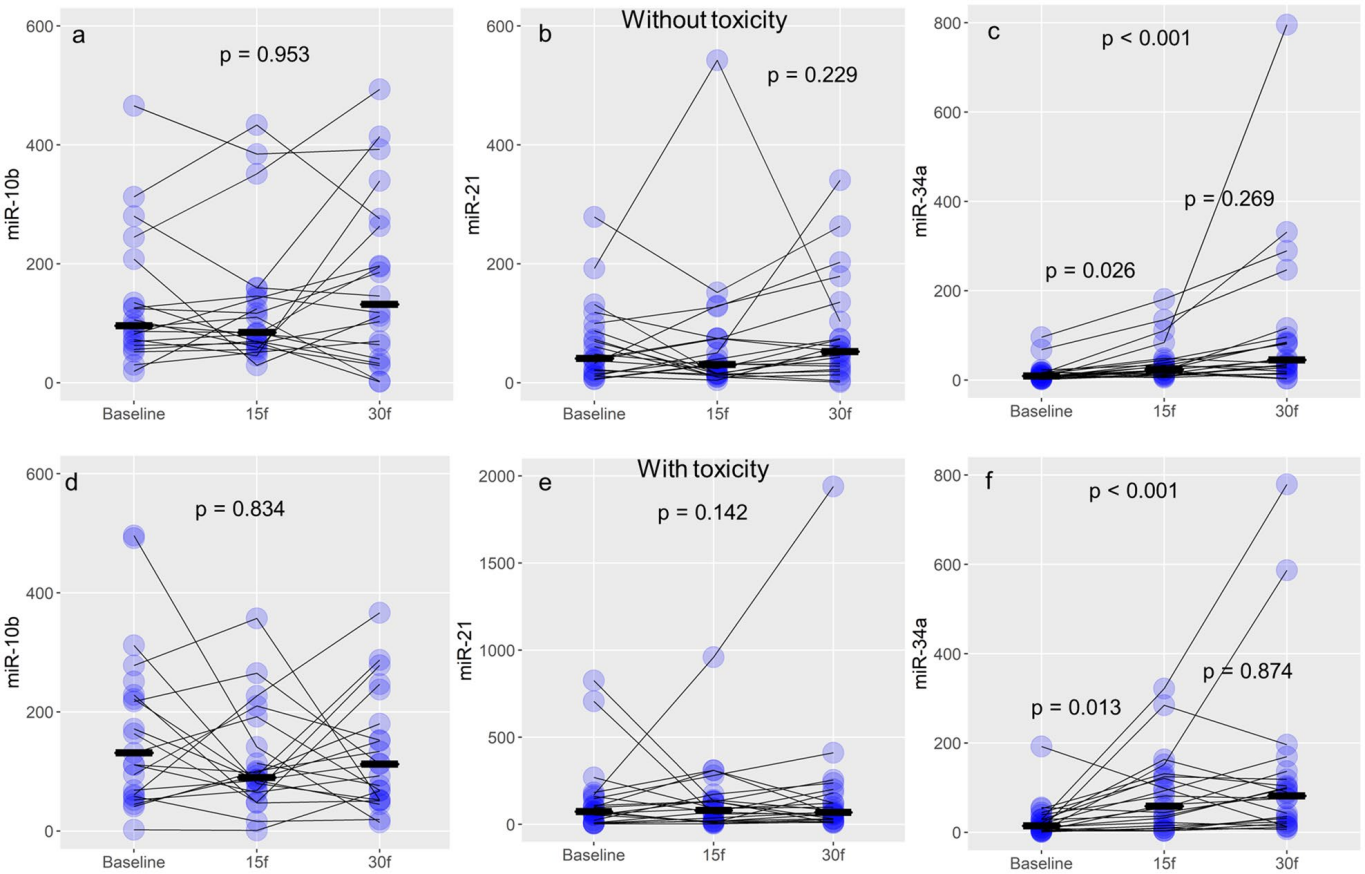
Changes of miR-10b/21/34a over time during the treatment. Violet dots represent each patient, while the black line represents the median value of expression (*RQ* relative quantity units) for miR-10b (a-without toxicity, and d-with toxicity), miR-21 (b-without toxicity, and e-with toxicity), and miR-34a (c-without toxicity, and f-with toxicity).

Within the group of patients without the side effects (N = 21) levels of miR-34a expression were significantly elevated at the 15th and 30th fraction of RT compared with baseline levels (p < 0.001 Friedman’s tests, Table 3, Fig. 3). Furthermore, the pairwise comparison has been performed among all RT fractions: baseline with 15th, baseline with 30th, and 15th with 30th. Significance values were adjusted using the Bonferroni correction method for multiple testing. MicroRNA 34a expression levels were significantly higher in 15th fraction compared with baseline (Wilcoxon’s test, p = 0.026, Fig. 3c), and at 30th fraction compared with baseline levels (Friedman’s test, p < 0.001, Table 3, Fig. 3c). Expression levels of miR-34a did not significantly differ between 15 and 30th fraction (Wilcoxon’s test, p = 0.269, Fig. 3c). It should be noted that median expression levels of miR-34a were the highest at 30th fraction. The situation was similar in the groups of patients with side effects of RT (N = 22). Levels of miR-34a were significantly different among baseline, 15th fraction, and 30th fraction (Friedman’s test p < 0.001, Table 3, Fig. 3f). At the 15th and 30th fraction, respectively, miR-34a levels were significantly higher than the baseline, but 15th and 30th fraction levels were not significantly different (Wilcoxon’s test, p = 0.013, p < 0.001, and p = 0.874, Fig. 3f).

### Correlational analysis

Significant positive correlations have been found among all the three miRNAs at all of the three investigated time points, indicating that all of the three miRNAs change and follow the same direction-profile in response to treatment in PBMCs (Table 4). The strongest correlation (with the highest correlational coefficient) was found between miR-10b and miR-21 at the 15th fraction within patients with toxicity (rho = 0.950, p < 0.001, Spearman’s test); miR-21 and miR-34a at the 30th fraction within patients without toxicity (rho = 0.856, p < 0.001, Spearman’s test); miR-21 and miR-34a at the 15th fraction within patients without toxicity (rho = 0.846, p < 0.001, Spearman’s test); miR-21 and miR-34a at the 15th fraction within patients with toxicity (rho = 0.807, p < 0.001, Spearman’s test).

**Table 4:** Correlations among miRNA-10b/21/34a expression levels.

Variable 1	Variable 2	N^a^	Time point	Toxicity	Correlational coefficient^b^	p value
miR-10b	miR-21	28	15th f	No	rho = 0.441	**0.019**
miR-10b	miR-34a	28	15th f	No	rho = 0.376	**0.049**
miR-21	miR-34a	28	15th f	No	rho = 0.846	**< 0.001**
miR-10b	miR-21	15	15th f	Yes	rho = 0.950	**< 0.001**
miR-10b	miR-34a	15	15th f	Yes	rho = 0.789	**< 0.001**
miR-21	miR-34a	15	15th f	Yes	rho = 0.807	**< 0.001**
miR-10b	miR-21	26	30th f	No	rho = 0.644	**< 0.001**
miR-10b	miR-34a	26	30th f	No	rho = 0.590	**0.002**
miR-21	miR-34a	26	30th f	No	rho = 0.856	**< 0.001**
miR-10b	miR-21	17	30th f	Yes	rho = 0.762	**< 0.001**
miR-10b	miR-34a	17	30th f	Yes	rho = 0.434	0.082
miR-21	miR-34a	17	30th f	Yes	rho = 0.770	**< 0.001**

^a^N-number of patients. ^b^Correlation coefficient. p values equal or less than 0.05 are significant (bold).

### Bioinformatics analysis

According to miRNet^32^, a miRNA-centric network visual analytics platform bioinformatics tool, there are 6 common target genes for miR-10b/21/34a: Breast cancer type 1 susceptibility gene (*BRCA1*), Kelch repeat and BTB domain-containing protein 6 (*KBTBD6*), MAP kinase-interacting serine/threonine-protein kinase 2 (*MKNK2*), peroxisome proliferator activated receptor alpha (*PPARA*), tropomyosin 1 (*TPM1*), and nuclear FMR1 interacting protein 2 (*NUFIP2*) which are parts of MAPK, insulin, dilated cardiomyopathy, PPAR, adipocyte, Fanconi anemia (FA), hypertrophic cardiomyopathy, and cardiac muscle contraction signaling pathways. Furthermore, we described genes involved in a response to irradiation.

## Discussion

In planning the postoperative radiotherapy for glioblastoma, after defining gross tumor volume (GTV), an isotropic margin of 2 cm is added to create clinical target volume (CTV), which is later modified according to the protocol (i.e., anatomical barriers). A margin of 3–5 mm is added to create planning target volume (PTV)^33^. A bigger target volume means a bigger irradiated brain volume and/or critical structures; this, in turn, correlates directly with the risk of brain injury and toxicities, respectively. Side effects of cranial irradiation of brain tumors are recognized as early or acute and late toxicity. According to literature, sometimes early delayed or subacute toxicity can also be observed and described. Acute toxicity may occur within a few hours or days after the first fraction of RT. Given that radiotherapy is administered concomitantly with temozolomide chemotherapy, it cannot be affirmed with certainty that certain acute side effects are a mere consequence of radiotherapy or chemotherapy. However, systemic side effects such as myelosuppression or diarrhea are thought to be a result of chemotherapy^16^. Stupp et al. reported that the most common non-hematological side effect during radiotherapy was fatigue in 26% of patients in the radiotherapy group and 33% in the radiotherapy + TMZ group^3^.

Although the mechanism underlying radiation toxicity is complex and remains partially unclear, there are numerous molecular and cellular mechanisms engaged in CNS toxicity. In vivo study has shown that radiation can induce cytokine response in the brain and an early acute pro-inflammatory gene expression^34^. Also, acute neurotoxicity is associated with the occurrence of late neurotoxicity^16^. In addition, as ionizing radiation damages cells mostly through free radicals, it is considered that late side effects of cranial irradiation may be the result of long-acting free radicals and reactive oxygen species (ROS), cytokines^35^, and miRNAs regulating oxidative stress signaling components^27^, amongst other biomolecules. MicroRNA-34a is involved in the overproduction of nicotinamide adenine dinucleotide phosphate oxidase 2 (NOX2) and other ROS-generating enzymes^36^, potentially influencing oxidative/antioxidative balance^36,37^. MicroRNA-34a also changes the expression profile of cytokines such as interleukin 6 (IL-6), and tumor necrosis factor alpha (TNFα), thus regulating the inflammatory response^27^. Besides that, it has been noticed that *IL-6* and transforming growth factor beta (*TGF-β*) gene expression levels change in PBMC in prostate cancer patients receiving RT^38^. MicroRNA-10b was shown to decrease radiation-induced apoptosis in glioblastoma cells^25^. MicroRNA-21 is perceived to be radiosensitive and is involved in the inflammatory response, as well^39^. The inflammatory response is also one of the major reactions to radiation-induced normal tissue injury^40^. It has been also shown that miR-10b and miR-21 co-inhibition can significantly decrease cell growth and invasion of U87MG glioblastoma cells, indicating their synergistic effect on their shared genes^26^. MiR-21 silences mRNAs whose protein products regulate the cell cycle, apoptosis, DNA damage repair, hypoxia, and is associated with normal tissue/tumor radiosensitivity and tumor radioresistance^41^. MicroRNA-34a induces radiosensitivity through the increase of apoptotic rates and inhibition of cell viability, by silencing Bcl-2, among others^42^. When Bcl-2 expression is lower after irradiation, apoptosis increases, and miR-34a radio-protective inhibitor decreases, which was shown on GB cells^27,42^. That in turn might increase radiation injury.

In this study, different statistical approaches have been used to shed light from different angles on the association between miRNA molecules and toxicity and grade over time. Toxicity rises during the time of radiation exposure, and it is a well-known fact, which is also in accordance with our results. MicroRNAs 10b and miR-21 increase was associated with a higher toxicity grade, which indicates that those two miRNAs might be promising indicators of toxicity grade, unlike miR-34a, which has a different role in regulating homeostasis after radiation exposure. MicroRNA 10b and miR-34a were significantly different between the groups with and without toxicity at the 15th fraction, but not at the 30th fraction, indicating that the most important events underlying the biological background of RT-induced toxicity might happen at this time point, in particular. MicroRNA-34a expression levels significantly increased after the combined treatment, and differed between 15th fraction of RT and baseline, and 30th fraction of RT and baseline, but not between 15 and 30th RT fractions, as expected. Earlier it has been shown that miR-34a might have the potential to be utilized as a dosimeter for radiation exposure (it raised with radiation dose). It should be kept in mind that therapeutic doses in these cases are fractionated, and patients each time receive the same, 2 Gy dose of radiation (which significantly reduces the risk of side effects of RT over time). The differences between the toxicity groups indicate that miR-34a has the potential to stratify patients into the low and high-risk groups for developing side effects of RT. Other studies have shown that miR-34a regulates biological processes underlying radiotoxicity such as cytokine production and vascular damage leading to fibrosis, and also DNA damage response^27^. Also, it has been shown that miR-34a level changes in T-lymphocytes were associated with time, dose, and mutation status of ataxia telangiectasia mutated (ATM) gene^43^, which is additional proof of its association with extreme individual radiosensitivity.

Correlational analysis has shown that each miRNA complements each other in the terms of response to radiation and that they could have a synergistic effect, or that they just work in some sort of combination in response to RT. Multiple miRNAs can also target the same gene, suggesting that a combination of those miRNA activities might determine the expression of a specific mRNA^44^. Their signaling pathways intersect, according to our bioinformatics analysis. Also, the results of bioinformatics analysis indicate that shared gene targets among the three investigated miRNAs, such as *BRCA1*. Protein BRCA1 promotes homologous recombination-mediated DSBs repair and represents a potentially good predictive molecule for response to RT^45^. Double-strand breaks are the most significant events in response to ionizing radiation exposure, which activates DNA damage repair machinery. KEGG pathway analysis has shown that targets of the three miRNAs are involved in the Fanconi anemia signaling pathway. Fanconi anemia is an autosomal recessive or X-linked genetic disorder characterized by chromosome fragility, congenital malformations, and cancer susceptibility. FA patients are usually extremely sensitive to irradiation and radiotherapy^46^. Tropomyosin gene silencing was associated with radioresistance of glioma cells^47^; higher *MKNK2* expression was detected in glioblastoma cells, compared with other brain tumor cell subtypes^48^. In the previous study by Kopcalic et al.^31^ miR-21 was shown to vary significantly between patients with and without radiotoxicity in prostate cancer patients who underwent three-dimensional (3D) conformal RT, contrary to the findings from this study. It should be mentioned that the study of Kopcalic et al.^31^ investigated prostate cancer acute genitourinary radiotoxicity and different radiation volumes and organs at risk (side effects were different). So, the utilization of each miRNA for the prediction of RT-induced side effects might vary among different types of malignancies. MiR-21 levels change in PBMCs in response to RT, and might be associated with organ-specific tumor or grade-in case of GB, or with a risk of acute radiation toxicity in the case of radiation treatment for PCa^31^. Still, larger cohorts are needed to confirm these findings as well as inter and intra-validation studies to investigate if microRNAs should be taken into consideration for further study and research as biomarkers for the prediction of side effects in patients with malignant neoplasms receiving radiotherapy.

The limitation of our study might be the relatively small number of patients who have been analyzed, but not the number of patients per group. The advantage of our study is that the group of patients analyzed in this study was relatively homogenous (all patients received the same treatment according to Stupp's protocol and target volume delineation was conducted by ESTRO-ACROP guidelines^33^ of target delineation of glioblastomas); there were no differences among patients grouped according to age or gender. Also, groups divided according to toxicity were equally distributed, 22 versus 21 patients). Because of the complex mechanisms and genetic/epigenetic events underlying toxicity in combined treatment, we cannot confirm with certainty whether microRNA changes are associated only to radiotoxicity. Moreover, microRNAs 10b/21/34a are mostly investigated in correlation with radiation exposure. Further investigation is necessary to distinguish the side effects of both treatment modalities and their correlation with microRNA expression.

One of the most important results of this study is that miR-34a might indicate which group of patients could be a candidate for dose escalation shortly, but a clinical study is needed to confirm the hypothesis which has resulted from this research. There are very limited number of studies investigating microRNA expression levels in GB patients in association with the side effects of RT and the change of their expression level. Each of the investigated miRNAs might have a special role in a cascade of radiation response signal transduction, which needs to be elucidated. Their expression level changes might be utilized for the prediction of side effects of RT shortly. This research also has the purpose to encourage further research in this field to find biomarkers for early prediction of side effects of RT, as well as of chemotherapy with TMZ, so clinicians can consider alternative treatment regimens, and take a step closer towards the prediction of individualized normal tissue radiosensitivity.

## Conclusion

According to the results of this study, miR-34a might be considered in the future as a factor for the prediction of toxicity at the 15th fraction of RT. In addition, miR-34a might be used for novel biomarkers approach panel that measures variations of miRNAs over time as indicators of acute side effects in GB patients. MicroRNA 10b might be used to predict toxicity and grade, while miR-21 can be used only for the prediction of the grade of toxicity within GB patients. According to miR-10b and miR-34a expression variations, the 15th fraction might be the key point for the prediction of response to RT. The findings of this research might represent significant progress towards the individualization of the treatment in patients with GB, which is a priority goal for researchers and neurooncologists. This concept is unique, suggesting that dynamic changes such as microRNA expression variations might be used as parameters for predicting radiosensitivity and response to RT to improve the quality of life in cancer patients. MicroRNA signatures at particular time points during the course of treatment might represent a significant advance in the field of cancer biomarker research shortly. This study represents an integrative approach of clinical data, therapy, molecular biology, and bioinformatics analysis, which are all necessary segments for future individualized treatment of patients, to discover the most efficient therapeutic regiment.

## Methods

### Study population

The study, which was designed as a prospective cohort study, investigated expression levels of 3 miRNA molecules-miR-10b/21/34a extracted from PBMCs of 43 patients diagnosed with glioblastoma treated at the Clinic of Neurosurgery, Neuro-Oncology Department, University Clinical Center of Serbia and at the Institute for Oncology and Radiology of Serbia, Department of Radiation Oncology, Serbia, starting from October 2017. The patients included in the study signed the informed consent. All methods were performed in accordance with the relevant guidelines and regulations and the study protocol was conformed to the ethical guidelines of the Declaration of Helsinki. After the surgery, patients continued RT in combination with TMZ, and adjuvant TMZ. Radiotherapy was planned with the 3D conformal or Volumetric Modulated Arc Therapy (VMAT) technique. Target volumes were delineated according to ESTRO-ACROP target delineation of glioblastoma^35^. Preoperatively and postoperatively, according to protocol, computerized tomography (CT) scan and/or magnetic resonance imaging (MRI) were obtained for all patients. All necessary laboratory tests for the hematological, renal, and liver function assessment were obtained, as well. The study protocol was approved by the Ethical Research Committee of the Faculty of Medicine, University of Belgrade (ethical board approval № 1322/X-39).

### Clinical parameters of patients and pathohistological characteristics

Clinical parameters-mean age at diagnosis, gender, pathohistological characteristics-IDH mutation status, histological subtype (glioblastoma, glioblastoma with primitive neuronal component, and giant cell glioblastoma) and presence of toxicity is shown in Table 1.

### Patients’ treatment

Fractionated RT started four to six weeks after the surgery, with 30 fractions at a dose of 2 Gy per fraction, and lasted for 6 weeks, with a total dose of 60 Gy. Concomitant therapy involved 75 mg/m^2^ TMZ, administered 7 days a week from the first to the last day of RT. Then, the patients went on a 4-week treatment pause, and then continued with adjuvant TMZ at 6 cycles, 5 days a week (every 28 days). During the initial cycle, the patients received 150 mg/m^2^ of TMZ, followed by a dose increase up to 200 mg/m^2^. Acute toxicities that are observed during the treatment are headache, nausea, vomiting, seizures, fatigue, somnolence, confusion, and agitation. Acute toxicity was measured and graded weekly according to the Common Terminology Criteria for Adverse Events (CTCAE), Version 5.0.

### Exclusion criteria

Patients with comorbidities such as respiratory and cardiovascular acute renal failure, acute surgical or other infectious conditions, or allergy to chemotherapy, as well as patients who received any hormone therapy (other than corticosteroid therapy, which is symptomatic therapy for GB patients) were excluded from the study.

### Isolation of peripheral blood mononuclear cells

Peripheral blood mononuclear cells were extracted from heparinized whole blood by centrifugation at 4 °C using Histopaque-1077 Sigma-Aldrich, a density gradient medium, according to the manufacturer’s manual.

### RNA extraction

The extraction and purification of miRNA molecules from PBMCs was performed with TRI Reagent (Sigma Aldrich) according to the manufacturer’s protocol (0.2 ml of chloroform and 0.5 ml of isopropanol per 1 ml of TRI reagent followed by 1 ml of 75% ethanol dissolved in nuclease-free water. The RNA samples were then quantified on BioSpec-nano (Shimadzu Corporation, Japan) spectrophotometer. The samples with an odds ratio of A260/280 nm between 1.7 and 2.1 were considered adequate for future analysis.

### Reverse transcription and real-time quantitative polymerase chain reaction (RT-qPCR)

Reverse transcription of miR-10b/21/34a molecules included specific TaqMan^®^MicroRNA assays with ID: 002218, 000397, and 000426 respectively (Applied Biosystems, Foster City, CA, USA), endogenous control, RNU6B (001093), and Taqman^®^MicroRNA Reverse Transcription Kit (Applied Biosystems by Thermo Fisher Scientific, Vilnius, Lithuania). For the reaction of reverse transcription, we used 10 ηg of total RNA. By the reaction of real-time quantitative polymerase chain reaction RT-qPCR using primers with loop (Stem-Loop RT-qPCR), cDNA molecules were amplified with TaqMan™ Universal Master Mix II, no UNG (Applied Biosystems, Life Technologies, Warrington, UK) on 7500 Real-Time PCR System (Applies Biosystems, Foster City, California, USA). Relative quantity (RQ) values were calculated and obtained through 7500 System SDS software (Applied Biosystems, Foster City, California, USA), by comparative delta delta Cycle threshold (ddCt) method by formula RQ sample = 2^−(dCt sample − dCt calibrator)^; dCt = Ct_miR-10b/21/34a_ − Ct_RNU6B._ All samples were normalized to endogenous control RNU6B, and calibrated to the sample with the lowest RQ value.

### Statistical analysis

Firstly, a linear mixed-effects model was used in the lme4 package for the R statistical computing environment (R Core Team, Vienna, Austria, 2019) for each individual in all investigated time points. Furthermore, we used Friedman’s Two-Way analysis adjusted by the Bonferroni correction for multiple pairwise tests and Wilcoxon signed-rank test with a continuous correction test (for comparison of repeated measures at three and two time-points, respectively), Spearman's rank correlation test (for correlational analysis between miRNA levels), Fisher’s exact test for the distribution of patients in specific groups, in IBM SPSS Statistics 22 (IBM Corporation, Armonk, NY, USA) statistical software.

### Bioinformatics analysis

To elucidate combinatorial action of the three miRNAs-miR-10b/21/34a, miRNet bioinformatics tool was used. Online tool miRNet is a miRNA-centric network visual bioinformatics platform that integrates data on miRNA interactions with genes and has the ability to look for targets if multiple miRNA enters in various diseases and tissues^32^. For the analysis of the common genes shared among all three miRNAs, input parameters were as following: Organism: Homo sapiens; ID Type: miRBase ID; Targets: Genes (miRtarBase v8.0). Finally, we investigated which signaling pathway are activated, according to Kyoto Encyclopedia of Genes and Genome (KEGG) database-KEGG PATHWAY, which represents a set of pathway maps with interaction, reaction and networks among molecules of the particular signaling pathway^33^.

## Data Availability

The authors declare that the data supporting the findings of this study are available within the article.
